# Does human papillomavirus cause human colorectal cancer? Applying Bradford Hill criteria postulates

**DOI:** 10.3332/ecancer.2020.1107

**Published:** 2020-09-21

**Authors:** Muhammad Usman, Yasir Hameed, Mukhtiar Ahmad

**Affiliations:** Department of Biochemistry and Biotechnology, The Islamia University of Bahawalpur, Bahawalpur, Pakistan

**Keywords:** human papillomavirus (HPV), colorectal cancer (CRC), PubMed, Bradford Hill criteria, pathogenesis

## Abstract

The role of human papillomavirus (HPV) in human colorectal cancer (CRC) has already been widely investigated worldwide with conflicting results. Although researchers have tried to establish the link between HPV and CRC through a statistical meta-analysis of the previous studies associating HPV with CRC, they failed to establish a more reliable link due to the shortcomings of the statistical meta-analysis. In the present study, we identified population-wide studies relating HPV with CRC through the PubMed search engine. Then, we examined the available data of HPV prevalence in CRC and normal/benign samples and applied the postulates of Bradford Hill criteria on the available evidence to investigate the association between HPV and CRC. The Bradford Hill criteria are very old, reliable and widely accepted for establishing a link between the cause and disease. In addition, to further enhance the reliability of the outcomes, we have also evaluated the methodologies of the previous studies to address the possibility of false-negative and false-positive results. After a careful evaluation of the extracted data against the postulates of Bradford Hill criteria, it was observed that none of the studies fulfil all the major postulates of Bradford Hill criteria for causation including temporality, consistency, biological gradient, experiment, coherence, specificity and analogy. Hence, no causal relationship has been suggested between HPV and CRC patients of the any included population. The results failed to prove the causal relationship between HPV and CRC and suggested HPV as a coparticipant in the pathogenesis of CRC.

## Introduction

Colorectal cancer (CRC), also known as large bowel cancer, is a cancer of the colon, rectum and appendix [1]. In general, it develops gradually within 10–15 years. Patients with ulcerative colitis are at a higher risk of developing CRC compared to the general population [2]. Globally, CRC is the third most commonly diagnosed cancer in men and the second most frequently reported cancer in women [3]. In the United States, it is estimated that 147,950 new cases and 5200 CRC related deaths will occur in 2020 [4]. CRC is more prevalent in developed countries accounting for 63% of cases of all the cancer cases. The high-risk hotspots of CRC include Europe, Australia and North America. In these areas, CRC is more prevalent amongst urban residents than the rural residents [5].

More than 100 subtypes of HPV have been reported in the medical literature until now, of which 40 subtypes are known to infect the genital epithelial cells [6]. At least 15 HPV subtypes including HPV 16, 18, 31, 35, 39, 45, 51, 52, 56, 59, 66, 68, 69, 73 and 82 are classified as high-risk subtypes due to the significant association with genital tract and non-genital tract malignancy [7].

It has already been acknowledged earlier that two HPV viral oncogenes (E6 and E7) contribute majorly to the development of HPV-induced CRC. Oncoprotein E7 inactivates hypophosphorylated retinoblastoma protein (pRB) by tightly binding with it, and then, the inactivation of pRB eventually results in the upregulation of p16INK4A. P16INK4A is a tumour suppressor protein that is involved in inhibiting the cyclin-dependent kinases 6 and 4, which regulates the G1 checkpoint of the cell cycle. The role of oncoprotein E6 is well recognised for functionally inactivating p53 protein which is a major regulator of the cell cycle [8]. In earlier studies, a reported inverse correlation between p53 mutations and HPV infection suggested that HPV targets and inactivates the p53, and in turn, the inactivated p53 significantly contributes to the development and progression of CRC by dysregulating various important pathways [9].

The first-ever study documenting the presence of HPV was conducted in 1990 by Kirgan *et al* [10] in the Czechoslovakia population. In that study, they detected the presence of HPV in a total of 73 paraffin-embedded CRC samples and 30 normal colon mucosal tissue samples using the Southern blotting technique. The results of their study revealed the presence of HPV in approximately 82% and 23% of CRC and normal controls, respectively.

Since then, numerous studies have been carried out worldwide for detecting HPV in CRC, and their outcomes were contradictory because they detected HPV in varying frequencies in different populations, i.e., from 0% [11, 12] to 100% [13].

In general, a statistical meta-analysis is usually preferred when establishing a correlation between the virus and disease as compared to the single study. This choice is based on the multiple advantages of the meta-analysis such as increased number of objects, greater diversity amongst the objects and conclusion with a high level of evidence over the individual single study, which has disadvantages like a small cohort of patients and conclusions with a low level of evidence. By keeping in view the inconsistencies in the HPV detection ratios in worldwide published studies, recently, researchers have analysed the previously published studies by the means of statistical meta-analysis to yield more useful pieces of information.

Previously, a statistical meta-analysis was performed to find out the causal relationship between HPV and CRC by Damin *et al* [14] of the available literature on HPV detection in CRC by searching various authentic research engines including Medline (PubMed), Embase (OVID) and Web of Science. They obtained more than 18 studies from different populations such as Europe, Asian and American. The results of their meta-analysis revealed that HPV infection significantly increased the risk of CRC development.

Similarly, Baandrup* et al* [15] performed another meta-analysis of the available literature reporting the association of HPV with CRC through various authentic research engines such as PubMed and Web of Science. They analysed 37 studies from American, Asia and Middle East populations. In their conclusion, they also reported a significant association between HPV and CRC.

Although evaluating the results of the previous studies documenting the role of HPV in the development of CRC through statistical meta-analysis was a better choice than generalising the results of an individual study, we did not consider statistical meta-analysis reliable to establish a causal relationship between HPV and the CRC development because of some serious limitations such as its inability to analyse the methodologies of the previous studies, so there is no way to evaluate the possibility of false-negative and false-positive results nor does statistical meta-analysis provide any information regarding the effect of heterogeneity-specific nature of the understudied populations on HPV detection. In addition, statistical meta-analysis results in publication biasness, where meta-analysis does not select studies with no results even though they contain valuable information.

By looking at the discrepancy in the outcomes of the previously published studies and significant shortcomings of the statistical meta-analysis, we performed the population-wide valuation of the results of the previous studies using the Bradford Hill criteria. These criteria are widely used and accepted worldwide over many years for establishing a causal relationship between a presumed cause and an observed effect on public health research [16].

In the course of evaluation, we analysed whether or not these studies fulfil all the postulates of Bradford Hill criteria to declare a causal relationship between HPV and CRC. In addition, we also evaluated the methodologies used by the previous studies to address the possibility of false-positive and false-negative results for better outcomes. The outcomes of the present study will help to establish a more reliable population-wide causal relationship between HPV and CRC and determine the more appropriate treatment strategies for CRC patients.

## Methodology

**Figure 1.**
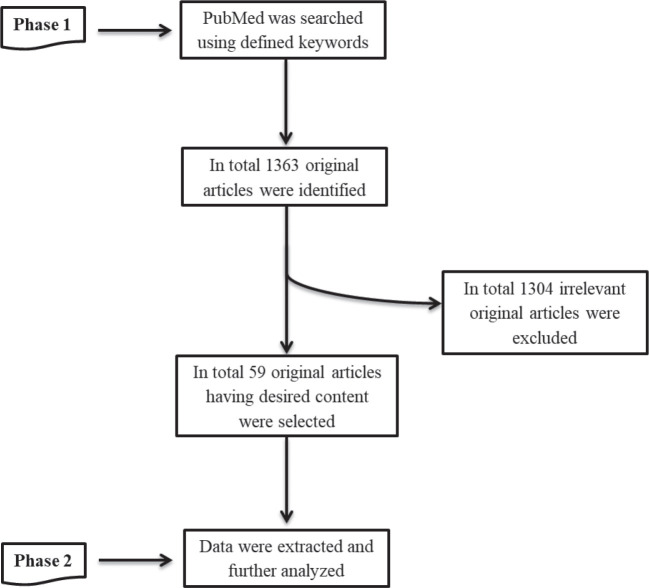
Overview of the methodology implemented during the present study.

In the present study, we implemented a two-phase methodology (Figure 1).

### Literature search

All the relevant articles associating HPV with CRC were identified through the PubMed search database using the keywords: ‘Colorectal Cancer’ and ‘Human Papillomavirus’. We also defined ‘Papillomaviridae’ and ‘Colorectal neoplasia’ as medical subject headings (MeSH) terms. MeSH terms and keywords were combined during the search process. All the literature works were searched available until March 2020, with the ‘Original Article’ filter. In total, 1,363 original articles were identified through the PubMed search engine.

### Relevant data extraction

From 1363 original articles, 59 relevant articles were identified having the desired information by initially reading the title, abstract and then complete article. Furthermore, a comprehensive table was constructed having all the required information from the selected relevant studies.

### Evaluation of the results using the postulates of Bradford Hill criteria

Based on the extracted data, all the identified studies were carefully evaluated using the following postulates:

**(1) Strength:** Larger the association, more probability of the causal relationship, **(2) temporality:** cause must lead to the induction of an effect. If the delay is expected between the cause and effect, then the effect has to occur after the delay, **(3) consistency:** different studies conducted by different researchers at different places with different sample sizes and reporting the similar results increase the chances of the causal relationship between the cause and effect, **(4) plausibility:** there should be plausible mechanism between the cause and effect, **(5) biological gradient:** greater response is produced by the causative agent in response to the greater exposure. However, in some cases, the effect can be triggered by the mere presence of the factor, whereas, in other cases, greater exposure can lead to lower effect as well, **(6) experiment:** the relationship between the cause and effect should be explained by the experiments, and the experiment should result in the reduction of effect when the causative agent is removed, **(7) coherence:** causal relationship should not conflict with already known literature about the disease or exposure, **(8) specificity:** causality is more likely if the effect has only one cause and **(9) analogy:** previous evidence of the association between the cause and effect should support the current statement for the causal relationship.

The assessment of each postulate was qualitative/descriptive, as there was an element of subjectivity in applying quantitative scoring. Evidence collected for each postulate is presented in Table 1 and the results section with a final judgment as to whether or not the viewpoint was fulfilled.

## Results

**Figure 2.**
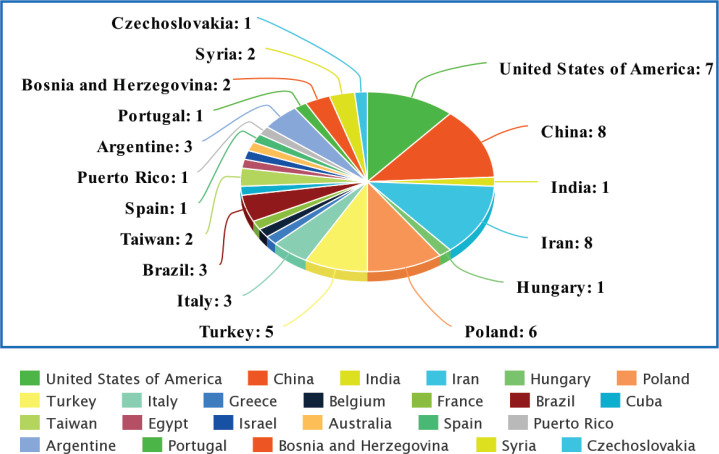
Comparison graph between the numbers (No.) of the studies carried out in each population on HPV in the CRC.

In total, 59 relevant original articles (Table 1; Figure 2) were found on PubMed which investigated the association of HPV with CRC in 24 different populations. Table 1 shows all these articles and contains essential information extracted from them including the details of the studied population, techniques used for HPV detection, name of the target gene, the identified strains of HPV, the most prevalent identified stain and number (No) of screened samples (normal, benign and diseased) with their respective identified population-wide positivity ratios.

Of all the 59 studies, in total, only 43 studies [12, 17–58] were the case–control studies, in which normal, benign and CRC samples were screened, whereas others were not.

The positivity ratio of HPV detection in CRC samples was varied population wise from 0% [11, 12, 17, 23, 26, 32, 41, 43, 45, 46, 50, 59] to 100% [13, 54] in all the 59 studies, whereas the positivity ratio of HPV detection in normal and adjacent/benign samples was varied from 0% [21, 22, 29, 30, 43, 49] to 84% (28) and 0% [17, 22, 26, 41, 42, 45, 46, 50, 52, 57] to 69.56% [54], respectively.

The results obtained after careful evaluation of the extracted data through the Bradford Hill criteria postulates showed that all the identified studies from various populations do not fulfil the major postulates including temporality, consistency, biological gradient, experiment, coherence, specificity and analogy. Hence, no causal relationship has been suggested between HPV and CRC patients of any included population.

Polymerase chain reaction (PCR) technique was employed by most of the studies [8, 10, 11, 13, 17-35, 37–49, 52, 54, 55, 57–91] to detect the presence of HPV in the normal, adjacent/benign and diseased samples using L1, E6 and E7 gene-specific primers which specifically target [6, 11, 16, 18, 31, 33, 35, 39, 40, 42, 45, 51–59] the subtypes of HPV, and from them, in addition, few studies also employed the second techniques including immunohistochemistry [67] and Southern blotting [13, 28] to validate their PCR results.

Few studies also used immunohistochemistry [31, 36, 51, 56], gene chip technology [53], Southern blotting [50] and *in situ* hybridisation [12] for the detection of HPV, and they did not validate their results through any other technique.

## Discussion

CRC is one of the most common types of cancer, which infects millions of people worldwide each year [92]. Although recent advancements in the diagnosis and treatment of the CRC have helped to manage the disease, the prevalence of CRC is still on the rise due to unknown underlying mechanisms [93].

To date, various individual studies have been carried out worldwide to find the state of association between HPV and CRC to further uncover the molecular pathways regulating CRC, but their results are conflicting. In addition, the statistical meta-analysis was also used by the researchers to analyse the previous individual studies for generating a more meaningful association between HPV and CRC, but, due to the shortcomings of statistical meta-analysis, researchers once again failed to establish a more reliable causal relationship between HPV and CRC.

In the present study, we evaluated the previous studies using a reliable, Bradford Hill criteria to find a causal relationship between HPV and CRC. In addition, we also evaluated the methodologies used by the previous studies to address the possibility of false-positive and false-negative results for better outcomes.

In total, 59 original articles were included in the present study. The HPV positivity ratio reported in these studies was varied between 0% [11, 12, 17, 23, 26, 32, 41, 43, 45, 46, 50, 59] and 100% [13, 54]. In most of the case–control studies [12, 17–27, 29–32, 35–57], the positivity ratio for HPV detection was higher in the cancerous samples as compared to the controls, whereas, in some studies [28, 33, 34, 58], HPV positivity ratio was higher in the controls as compared to the CRC samples. Possible reasons for such population-specific inequalities in HPV detection could be non-modifiable factors such as genetic makeup and socially controllable factors such as health-seeking behaviour and differential access to the health facilities. The HPV aetiology in CRC has been discussed population wise as follows:

### HPV aetiology in CRC patients of the United States of America (USA)

In the USA, a total of seven studies [11, 21, 22, 26, 33, 51, 64] including four [21, 22, 26, 33] case–control studies have been conducted so far to find out the association between HPV and CRC. These studies used immunohistochemistry and PCR techniques with primers specific for E6, E7 and L1 regions of the viral genome for HPV detection and documented differential HPV detection positivity ratios varying between 0% [21, 22] and 82% [51] in CRC samples, whereas 8% [33] and 38% [33] in normal and adjacent/benign controls, respectively. In the US population, HPV strain 16 was the most frequently reported strain (Table 1).

### HPV aetiology in CRC patients of China

In the Chinese population, a total of *n* = 8 [24, 29, 42, 47–49, 53, 56] case–control studies have carried out up to now reporting an association between HPV and CRC. All of these studies utilised gene chip technology, immunohistochemistry and PCR techniques for the detection of HPV using E6, E7 and L1 region-specific primers and documented varying HPV detection positivity ratios ranging from 21.9% [47] to 73% [42] in CRC samples. On the other side of the coin, they also documented 0% [29, 49] HPV detection positivity in normal and 0% [42]–29.7% [24] in adjacent/benign controls. Hence, their results revealed the higher HPV detection positivity ratios in CRC samples as compared to the normal and adjacent/benign controls. HPV strains 16, 6 and 33 were the most commonly identified strains in the Chinese population (Table 1).

### HPV aetiology in CRC patients of India

In India, a single case–control study [27] has been reported so far to find out the association between HPV and CRC. They analysed 93 CRC samples and 30 adjacent/benign controls using L1 gene-specific primers through the PCR technique and identified 6% HPV detection positivity ratio in normal and 36.5% positivity ratio in the CRC samples. The HPV18 was the most prevalent identified strain in the Indian population (Table 1).

### HPV aetiology in CRC patients of Iran

In Iran, so far, *n* = 8 studies [17, 30, 37, 39, 43, 75, 79, 80] have been reported to elaborate the HPV aetiology in CRC. From them, only *n* = 3 studies [30, 39, 43] were the case–control studies. All these studies employed the PCR technique for HPV detection using primers specific for E6, E7 and L1 region of the viral genome. In this population, HPV detection positivity ratios were reported in varying frequencies ranging from 0% to 35% in CRC samples, whereas 0% [30, 43]–1.25% [39] and 0% [17, 43]–5.7% [30] in normal and adjacent/benign samples, respectively. The HPV strains 16, 18, 55 and 56 were the most commonly identified strains in the Iranian population (Table 1).

### HPV aetiology in CRC patients of Hungary

In Hungary, a single study [13] has been reported to date relating HPV with CRC. They screened only 01 CRC sample using Southern blot hybridisation techniques, and it was positive for HPV 16 (Table 1).

### HPV aetiology in CRC patients of Poland

To date, a total of *n* = 6 studies [20, 35, 41, 54, 59, 74] including *n* = 2 [41, 54] case–control studies have been carried out in Poland to determine the association between HPV and CRC. HPV detection was done in these studies through the PCR technique using primers specific for E6, E7 and L1 region of the HPV genome. They documented the HPV detection positivity ratios with varying frequencies ranging from 0% [41, 59] to 100% [54] in the CRC samples, whereas 28% [35] in normal samples and from 0% [41] to 69.5% [54] in adjacent/benign samples. HPV 16 was the most commonly identified strain in the Poland population (Table 1).

### HPV aetiology in CRC patients of Turkey

A total of five studies [18, 34, 40, 46, 65] have been reported in Turkey until now relating HPV with CRC including three [18, 40, 46] case–control studies. They utilised PCR technique for the detection of HPV using L1 region-specific primers and identified HPV detection positivity ratios with varying frequencies ranging from 0% [46] to 82.14% [40] in CRC samples and 0% [46] to 32% [40] in adjacent/benign. The HPV strains 18 and 33 were the most commonly identified strains in the Turkish population (Table 1).

### HPV aetiology in CRC patients of Italy

So far, a total of *n* = 3 studies [44, 71] including a single case–control study [44] have been reported in Italy to demonstrate the relationship between HPV and CRC. They utilised the PCR technique for the detection of HPV using E6, E7 and L1 region-specific primers and identified HPV detection positivity ratios with varying frequencies ranging from 15.8% [44] to 33.3% [71] in CRC, whereas 8.8% [44] in normal samples. In Italian populations, HPV strains 16 and 18 were the most frequently detected strains (Table 1).

### HPV aetiology in CRC patients of Greece

Until now, only a single study [36] has been carried out in Greece to elaborate the association between HPV and CRC. In this study, a total of 60 CRC samples were analysed using the immunohistochemistry technique, and 26.6% HPV positivity ratio was documented (Table 1).

### HPV aetiology in CRC patients of Belgium

In Belgium, only a single study [8] has been carried out so far to find the association between HPV with human CRC. In this study, a total of 232 samples were screened using the PCR technique with specific primers targeting the L1 and E6 genes of the HPV genome, and their results revealed a 14.2% HPV detection positivity ratio (Table 1).

### HPV aetiology in CRC patients of France

Until now, in France, a single study [45] has been carried out to illustrate the association between HPV and CRC. In total, 217 CRC and adjacent/benign samples were screened using PCR, and HPV was not detected in any of the CRC or control samples (Table 1).

### HPV aetiology in CRC patients of Brazil

A couple of case–control studies [25, 52, 55] has been carried out in Brazil so far to analyse the association between HPV and CRC. They utilised PCR with primers specifically targeting the L1, E6 or E7 region of the viral genome and documented HPV detection positivity ratios with varying frequencies ranging from 13% [52] to 63.9% [25] in CRC samples, whereas 19.4% [25] in normal and 0% [52]–50% [25] in adjacent/benign controls. In the Brazilian population, HPV16 was the most commonly identified strain (Table 1).

### HPV aetiology in CRC patients of Cuba

Up until now, only a single case–control study [57] relating HPV with CRC has been reported in Cuba. They analysed 42 CRC and 21 adjacent/benign controls using PCR with primers specifically targeting the E6 and E7 regions of the HPV genome and documented 35.7% HPV detection positivity ratio in CRC samples only (Table 1).

### HPV aetiology in CRC patients of Taiwan

A couple of studies [28, 67] including a case–control [28] study has been carried out so far in Taiwan to find the association between HPV and CRC. They utilised Southern blotting and PCR for HPV detection and found a varying frequency of HPV detection ranging from 16% [67] to 53% [28] in CRC, whereas 0% in control samples. HPV 16 and 18 were the most commonly identified strains in the Taiwan population (Table 1).

### HPV aetiology in CRC patients of Egypt

A single case–control study based on HPV aetiology in CRC has been reported in Egypt so far. They examined 40 CRC as well as adjacent/benign controls using PCR and *in situ* hybridisation technique and documented 15% and 25% HPV detection positivity ratios in CRC and adjacent/benign controls, respectively (Table 1).

### HPV aetiology in CRC patients of Israel

In Israel, there has been a single case–control study [26] carried out so far to determine whether the HPV is associated with CRC or not. They analysed 106 CRC and 30 adjacent/benign controls for HPV detection using PCR and documented a 0% HPV detection positivity ratio (Table 1).

### HPV aetiology in CRC patients of Australia

Until now, a single study [12] has been carried out in Australia to demonstrate the relationship between HPV and CRC. They screened only 9 CRC samples using *in situ* hybridisation technique, and the presence of HPV was not detected in any of the sample (Table 1).

### HPV aetiology in CRC patients of Spain

Only a single case–control study [26] has been reported until now in Spain to relate HPV with CRC. In this study, a total of 100 CRC and 30 adjacent/benign controls were analysed using PCR with primers specific for the L1 region of the viral genome, and they observed no HPV marker (Table 1).

### HPV aetiology in CRC patients of Puerto Rico

A single case–control study [19] has been carried out in Puerto Rico so far to relate HPV with CRC. In total, 45 CRC and 36 normal samples were analysed in this study using the PCR technique, and they observed 42% and 2.8% HPV detection positivity ratio in CRC and normal controls, respectively (Table 1).

### HPV aetiology in CRC patients of Argentine

So far, in total, three studies [38, 58, 84] including a single case–control study [38] have been reported in Argentine relating HPV with CRC. All the studies utilised PCR with primers specifically targeting the L1 region of the viral genome and documented HPV detection positivity ratios with varying frequencies ranging from 0% [23] to 74% [38] and 33% [38] in adjacent/benign samples (Table 1), respectively.

### HPV aetiology in CRC patients of Portugal

Only a single study [32] has been carried out in Portugal to date to relate the HPV with human CRC. In total, 144 CRC samples were screened in this study using PCR with primers specific for the E7 region of the viral genome and showed a 0% HPV detection (Table 1).

### HPV aetiology in CRC patients of Bosnia and Herzegovina

A total of two studies [72, 94] have been reported in Bosnia and Herzegovina so far relating HPV with the CRC. For the detection of HPV, both the studies utilised PCR with specific primers targeting the E6 region of the viral genome and documented the presence of HPV with varying frequencies ranging from 50% (72) to 80% [94] in CRC samples. The most frequently identified strain in Bosnia and Herzegovina was HPV 16 (Table 1).

### HPV aetiology in CRC patients of Syria

So far, in Syria, the association between HPV and CRC has been accessed by only two studies [31, 70]. These studies utilised immunohistochemistry and PCR techniques for HPV detection with primers specific for L1, E6 and E7 regions of the viral genome and documented HPV detection positivity ratios in varying frequencies ranging from 36.2% [31] to 53.8% [70] in CRC samples (Table 1).

### HPV aetiology in CRC patients of Czechoslovakia

To date, a single case–control study [50] based on HPV aetiology in CRC has been carried out in Czechoslovakia. A total of 13 cancerous and 10 adjacent/benign samples were analysed in this study using the Southern blot hybridisation technique, and they documented 0% HPV positivity ratio in CRC and adjacent/benign samples (Table 1).

The careful evaluation of the results of identified studies through Bradford Hill criteria showed that all the studies failed to fulfil the major postulates including strength, temporality, consistency, biological gradient, experiment, coherence, specificity and analogy. Hence, we suggested that HPV acts as a coparticipant in the development of CRC rather than having a causal relationship that might combine with the other viruses, such as human immunodeficiency virus and hepatitis C virus and other factors including genetic abnormalities, smoking, alcohol consumption and obesity and may increase a person’s risk of developing CRC by affecting the body’s immune system.

However, limitations and some of the major issues related to methodologies used in the included studies have been discussed as follows.

### Possible causes of the false-negative results

Some studies failed to detect HPV presence in any of the cancerous or normal controls that they were investigating. How we can be sure that negative results for HPV detection in the investigating samples are not due to the poor quality of the extracted DNA? Many studies utilised positive control to avoid such situations [17, 30, 39, 43, 80], but few studies [18, 24, 34, 65] did not use positive control, so there is no way to confirm their negative results. The choice of inappropriate primers could lead to false-negative results, for example, primers that target L1 region of the HPV genome may be unreliable for the detection of HPV in tissues of advanced carcinomas, as L1 and E1 regions may be lost during the integration of viral genome into the host genome, whereas the E6/E7 regions remained consistently present, so this is the plausible explanation for the completely negative results of the studies [17, 26, 41, 43, 46].

### Possible causes of the false-positive results

Most of the studies that we summarised used PCR technique [8, 13, 18-20, 24, 25, 27-31, 34–40, 42, 44, 47–50, 52, 54, 55, 57, 58, 64–66, 70-72, 74, 75, 77, 79, 80, 84] for the detection of HPV, and none of them utilised second technique to confirm their positive results PCR, except one study [58] which utilised immunohistochemistry. The results of their second technique have deviated from the first one. The expression of the p16, p53 and some other genes could be used as a surrogate biomarker in HPV-infected CRC patients. Along with HPV detection, these surrogate biomarkers were also analysed by some studies [20, 27, 37, 47, 75] to further validate their findings, of which the studies of Laskar *et al* [27] and Karbasi *et al* [75] have validated their findings by analysing these surrogate biomarkers, whereas the other studies [20, 37, 47] were failed to validate their findings the surrogate biomarkers. Such deviations in the results of previous studies raise a big question mark on the selection of appropriate technique and their sensitivities.

### Comparison of normal, benign and malignant samples

The case–control studies are necessary to establish a causal relationship between the causative agent and the disease. Some of the studies that we summarised analysed only the CRC samples [8, 11, 13, 59, 64, 65, 67, 71, 74, 75, 79, 80, 84] and did not allow us to compare their results with normal or adjacent/benign controls. However, most of the studies also analysed the normal and adjacent/benign tissues along with CRC samples, and the comparison of their results demonstrated that HPV detection positivity ratios in CRC samples were higher in [12, 17, 27, 29–32, 35–57] studies, whereas lower in [28, 33, 34, 58] studies as compared to the normal and adjacent/benign controls. However, none of the studies has reported the association of HPV with specific CRC subtype and histologic grade.

## Conclusion

The results of this comprehensive review are controversial. They failed to prove the causal relationship between HPV and CRC, rather suggesting that it is a coparticipant in the pathogenesis of CRC. However, due to various limitations of the methodologies used by the previous studies to detect the presence of HPV in CRC, additional experiments are required to prove the HPV aetiology in CRC.

## List of abbreviations

CRC = colorectal cancer

E2 = envelope gene 2

E6 = envelope gene 6

E7 = envelope gene 7

TP53 = tumour suppressor protein 53

MeSH = Medical Subject Headings

## Figures and Tables

**Table 1.** Summary of the detection of HPV types and positivity rate in normal and CRC samples relative to the different selected articles.

Studied Population	Technique used for the viral genome detection	Target gene	Identified strain	Most prevalent identified strain	No. of normal samples screened	No. of normal samples positive for HPV	Percentage positivity of HPV for normal samples	No. of adjacent or benign samples screened	No. of adjacent or benign samples positive for HPV	Percentage positivity of HPV for adjacent or benign samples	No. of total screened CRC samples	No. of colorectal cancer samples positive for HPV	Percentage positivity of HPV for colorectal cancer	References
United States	PCR	L1	16, 18	--	0	0	0%	0	0	0%	50	0	0%	[11]
	PCR	L1	6, 11, 16, 18, 33	--	24	2	8%	21	8	38%	38	13	32%	[33]
	Nested PCR	L1, E6	16, 18, 45	16	10	0	0%	0	0	0%	55	28	51%	[21]
	PCR	L1	--	--	0	0	0%	30	0	0%	73	0	0%	[26]
	Real-time PCR	E6, E7	16, 18. 31, 33, 45	--	250	0	0%	254	0	0%	0	0	0%	[22]
	Immunohistochemistry	--	--	--	0	0	0%	0	0	0%	11	9	82%	[51]
	Real-time PCR	L1, E7	16	16	0	0	0%	0	0	0%	555	11	2%	[64]
China	PCR	E6, E7	6, 11, 16, 18	16	0	0	0%	37	11	29.7%	70	37	52.9%	[24]
	PCR	L1	6, 11, 18 33	6, 33	16	0	0%	10	1	10%	46	20	43.47%	[49]
	PCR	L1	16	16	0	0	0%	32	1	3.1%	32	7	21.9%	[47]
	PCR	E7	16	16	0	0	0%	82	4	4.87%	82	42	51.21%	[48]
	PCR	L1	16, 18, 51, 59	16	32	0	0%	0	0	0%	96	28	29.1%	[29]
	PCR	L1	--	--	0	0	0%	75	0	0%	75	55	73%	[42]
	Gene chip technology and immunohistochemistry	--	6, 11, 16, 18, 31, 33, 35, 39, 42, 43, 44, 45, 51, 52, 53, 56, 58, 59, 66, 68, 73, 83 and MM4,	16	0	0	0%	100	29	29%	95	46	48.4%	[53]
	Immunohistochemistry	--	6, 11, 16, 18, 31, 33, 42, 51, 52, 56, 58	--	0	0	0%	47	0	0%	47	15	31.9%	[56]
India	PCR	L1	16, 18	18	0	0	0%	30	6	20%	93	34	36.5%	[27]
Iran	PCR	L1	16, 18	18	0	0	0%	0	0	0%	60	21	35%	[37]
	PCR	L1	--	--	50	0	0%	8	0	0%	42	0	0%	[43]
	PCR	L1	--	--	0	0	0%	0	0	0%	100	1	1%	[80]
	Nested PCR	L1	11, 18, 31, 45	18	80	1	1.25%	0	0	0%	80	5	6.25%	[39]
	Nested PCR	L1	--	--	0	0	0%	60	0	0%	70	0	0%	[17]
	Nested PCR	L1, E6, E7	16, 18	16	0	0	0%	0	0	0%	38	12	31.5%	[75]
	PCR	L1, E6	16, 18	16	70	0	0%	70	4	5.7%	70	2	2.85%	[30]
	Qualitative real-time PCR	L1	51, 56, 16, 31, 33, 8, 39, 45	51, 56	0	0	0%	0	0	0%	84	19	22.6%	[79]
Hungary	PCR and Southern blotting	--	--	16	0	0	0%	0	0	0%	1	1	100%	[13]
Poland	PCR	--	--	--	--	--	28%	--	--	56%	--	--	67%	[35]
	PCR	E6, E7	--	--	0	0	0%	0	0	0%	186	0	0%	[59]
	Real-time PCR	L1	--	--	0	0	0%	10	0	0%	40	0	0%	[41]
	PCR	--	16, 18	16	0	0	0%	0	0	0%	50	10	20%	[74]
	Real-time PCR	6, 11, 16, 18	11	--	0	0	0%	23	16	69.56%	1	1	100%	[54]
	Nested PCR	L1, E6	16, 18	16	0	0	0%	0	0	0%	120	3	2.5%	[20]
Turkey	PCR	L1	6, 11, 16, 18, 33	18, 33	0	0	0%	0	0	0%	53	43	81.2%	[65]
	Nested PCR	L1	--	16	0	0	0%	22	1	4.5%	100	4	4%	[34]
	PCR	--	--	18, 33	0	0	0%	56	18	32%	56	46	82.14%	[40]
	PCR	L1	--	--	0	0	0%	49	0	0%	106	0	0%	[46]
	PCR	L1	--	16,18	0	0	0%	0	0	0%	93	3	3.2%	[18]
Italy	PCR	L1, E6, E7	6, 11, 16, 18, 31, 33, 43, 56, 58, 66	18	0	0	0%	0	0	0%	66	22	33.3%	[71]
	PCR	L1	16, 18, 33, 58	58	0	0	0%	57	5	8.8%	57	9	15.8%	[44]
	PCR	E2, E4, E5, E6, E7	6, 16, 26, 39, 53	16	0	0	0%	0	0	0%	65	11	16.9%	[77]
Greece	Immunohistochemistry	L1	1, 6, 11, 16, 18 31	16,18	0	0	0%	0	0	0%	60	16	26.6%	[36]
Belgium	PCR	L1, E6	6, 11, 16, 18, 31, 35, 39, 42, 43, 44, 45, 51, 52, 66	16	0	0	0%	0	0	0%	232	33	14.2%	[8]
France	Real-time PCR	E6, E7	6, 11, 26, 40, 43, 44, 53, 54, 66, 70, 71, 73, 16, 18, 31, 33, 35, 39, 45, 51, 52, 56, 58, 59, 68, 69, 82	--	0	0	0%	217	0	0%	217	0	0%	[45]
Brazil	Nested PCR	L1, E6, E7	16, 18, 31	16	72	14	19.4%	72	36	50%	72	46	63.9%	[25]
	PCR	L1	6, 11, 16, 18, 31, 33, 34, 35, 39, 40, 42, 43, 44, 45, 51, 52, 54, 56 58	16	0	0	0%	65	5	7.7%	79	36	45.5%	[55]
	PCR	L1	16, 18, 26, 31, 33, 35, 39, 45, 51, 52, 53, 56, 58, 59, 66, 68, 70, 73, 82, 6, 11, 40, 40, 42, 43, 44, 54, 61, 62, 67, 81, 83, 89	16	0	0	0%	105	0	0%	92	12	13%	[52]
Cuba	PCR	E6, E7	16, 18, 31, 33, 45, 52, 58	16	0	0	0%	21	0	0%	42	15	35.7%	[57]
Taiwan	PCR and Southern blotting	--	18	18	19	16	84%	0	0	0%	19	10	53%	[28]
	Nested PCR	L1, E6	16	16	0	0	0%	0	0	0%	69	11	16%	[67]
Egypt	Nested PCR and *in situ* hybridisation	--	16,18	18	0	0	0%	40	10	25%	40	6	15%	[58]
Israel	PCR	L1	--	--	0	0	0%	30	0	0%	106	0	0%	[26]
Australia	*In situ* hybridisation	--	6, 11, 16, 18	--	0	0	0%	0	0	0%	9	0	0%	[12]
Spain	PCR	L1	--	--	0	0	0%	30	0	0%	100	0	0%	[26]
Puerto Rico	Nested PCR	L1, E2	16	16	36	1	2.8%	0	0	0%	45	19	42%	[19]
Argentina	Nested PCR	L1	6, 16, 18, 33	16	0	0	0%	30	10	33%	54	40	74%	[38]
	PCR	L1	16, 18, 31, 33	--	0	0	0%	0	0	0%	7	0	0%	[23]
	Nested PCR	L1	16, 18, 31, 66	16	0	0	0%	0	0	0%	75	33	44%	[84]
Portugal	Real-time PCR	E7	16	--	0	0	0%	0	0	0%	144	0	0%	[32]
Bosnia and Herzegovina	PCR	E6	16, 18, 31, 35, 39, 45, 51, 52, 56	16	0	0	0%	0	0	0%	106	85	80%	[72]
	PCR	E6	16, 18, 31, 35, 45, 51, 52	16	0	0	0%	0	0	0%	106	53	50%	[94]
Syria	PCR	E6, E7, L1	16, 18, 31, 33, 35	16, 33, 18, 35, 31	0	0	0%	0	0	0%	78	42	53.8%	[70]
	Immunohistochemistry	E6, E7, L1	16, 18, 31, 33, 35, 45, 51, 52 58.	--	0	0	0%	0	0	0%	102	37	36.2%	[31]
Czechoslovakia	Southern blotting	--	2, 6, 16, 18	--	0	0	0%	10	0	0%	13	0	0%	[50]

PCR = Polymerase chain reaction.
